# Six Steps to Squats: Design, Feasibility, and Baseline Results of a Pilot Functional Rehabilitation Protocol After Orthopaedic Trauma

**DOI:** 10.7759/cureus.111050

**Published:** 2026-06-17

**Authors:** Ameen Suhrawardy, Ahmad Almaat, Omar Abdalla, Haytham Alqasmi, Alexa Jaume, Pranav Khambete, Manar Chebli, Bashar Jawich, Andreea Geamanu, Rahul Vaidya

**Affiliations:** 1 Department of Orthopaedic Surgery, Detroit Medical Center, Detroit, USA; 2 Department of Orthopaedic Surgery, Wayne State University School of Medicine, Detroit, USA

**Keywords:** functional rehabilitation, joint flexion, lower extremity fracture, orthopaedic trauma, pilot study, range of motion, rehabilitation protocol, squat assessment, weight-bearing

## Abstract

Background and objective

Functional, multi-joint assessments provide more insight into orthopaedic recovery than isolated range-of-motion or time-based metrics. The squat requires coordinated hip, knee, and ankle motion and reflects mobility, strength, and neuromuscular control. Squat-based assessments have shown value in anterior cruciate ligament injury, osteoarthritis, and arthroplasty populations, but no standardized squat-focused protocol exists for orthopaedic trauma rehabilitation. This article describes the Six Steps to Squats protocol, a structured, progressive squat-based rehabilitation and assessment program for isolated lower extremity fractures; evaluates the feasibility of administering it in a Level 1 trauma clinic; and reports baseline squat mechanics in the first enrolled cohort. Comparative outcomes of this ongoing pilot study will be reported upon completion.

Methods

This prospective comparative pilot study enrolled adults recovering from isolated lower extremity fractures. Patients were assigned to an experimental group if designated weight-bearing as tolerated (WBAT) within the prior 1-2 months or to a control group if 4-12 months post-WBAT. Exclusion criteria included prior lower extremity surgery, multiple injuries, arthroplasty, inflammatory arthritis, paraplegia, BMI >45, or more than three active comorbidities. The Six Steps to Squats protocol consists of six progressive phases targeting ankle mobility, hamstring flexibility, squat range of motion, unilateral flexion, assisted descent, and full unassisted squat ascent, with a standardized exercise prescription and defined graduation criteria for each step. Baseline assessments were supervised by trained research staff; the home program was supported by a patient guide with video demonstrations and monitored through monthly clinic visits and biweekly telephone calls. Hip, knee, and ankle flexion angles were measured using goniometry and digital image analysis during assisted and unassisted squats. Functional status was assessed with the SF-36, administered before the squat assessment.

Results

Twelve patients were enrolled (seven control and five experimental). Mean baseline unassisted flexion angles (control vs experimental) were 104.4° ± 19.1° vs 112.5° ± 25.7° at the hip, 95.5° ± 26.8° vs 88.5° ± 36.3° at the knee, and 98.6° ± 10.5° vs 100.8° ± 5.3° at the ankle. Assisted squat flexion angles averaged 119.2° ± 12.4° vs 111.0° ± 21.9° at the hip, 112.2° ± 26.5° vs 109.6° ± 40.6° at the knee, and 98.2° ± 8.8° vs 94.8° ± 5.7° at the ankle. All patients successfully rose from an unassisted squat, all 12 completed the full baseline assessment within a single routine clinic visit, and no adverse events occurred during testing. Preliminary subgroup analysis suggested reduced multiplanar flexion among patients with bimalleolar ankle fractures treated with open reduction and internal fixation.

Conclusion

The Six Steps to Squats protocol and its assessments proved feasible to administer in a Level 1 trauma clinic, and baseline data showed wide variability in squat mechanics after lower extremity fracture. Ongoing enrollment and follow-up of the full planned cohort will evaluate its effects on joint biomechanics, patient-reported outcomes, and long-term functional performance.

## Introduction

Musculoskeletal trauma contributes substantially to global disability, leading to long-term pain, impaired mobility, and loss of function. Current rehabilitation assessments often rely on static or time-based measures, which may not fully capture dynamic neuromuscular recovery [1]. Functional performance assessments, particularly those involving compound movements, are increasingly recognized for their utility in gauging readiness to return to activity and in identifying residual deficits [2]. Among these, the squat, and specifically the full-flexion squat, stands out as one of the most comprehensive and foundational human movements. The squat demands synchronized engagement of bilateral hips, knees, and ankles with adequate range of motion (ROM), strength, proprioception, and ligamentous stability of each joint. This makes it particularly suitable for assessing composite lower-extremity strength, balance, and neuromuscular control in a dynamic, weight-bearing environment [2-4].

The literature supports the squat as both a screening and rehabilitation tool. Once considered potentially harmful, deep or full-flexion squats are now accepted as safe and even therapeutic when performed correctly [3,5]. They promote ligament stability and quadriceps activation without compromising joint integrity [3,6], and structured programs show that they may enhance articular health [5]. In athletic training, squat assessments are widely used to detect biomechanical asymmetries and mobility deficits [2,4,7,8]. The deep overhead squat, a core element of the Functional Movement Screen (FMS), highlights deficiencies in bilateral mobility and postural control, and while the FMS has limitations in predicting injury, the squat component reliably reflects movement quality [2,4,8].

Despite the squat's ability to capture comprehensive lower-extremity function, its use as a functional benchmark or assessment tool in orthopaedics has been investigated only to a limited extent. In anterior cruciate ligament (ACL) reconstruction rehabilitation, squat-based tests reveal neuromuscular deficits that may persist despite normalized strength or completion of rehabilitation timelines, with approximately 45% of patients, six months postoperatively, failing single-leg squat tests [9]. Other studies have shown that squat mechanics offer critical insight into residual dysfunction missed by standard assessments, such as deficiencies in bilateral postural control during the squat after reconstruction and dynamic knee valgus with subsequent strain on the ACL due to pelvic drop during squat movement [10-12].

In patients with knee osteoarthritis (OA), structured squat-based rehabilitation programs have been shown to improve functional outcome measures [13]. Sit-to-stand variants of the squat are widely used in clinical settings to evaluate lower-extremity function and strength in older adults or individuals with musculoskeletal impairments [14]. These tests correlate with independence and real-world mobility, further supporting the functional value of the squat movement pattern. In patients with nonarthritic hip pathology such as femoroacetabular impingement or gluteal tendinopathy, single-leg squat and step-down tests are validated tools for guiding therapy, tracking clinical progress, and evaluating neuromuscular control, particularly for poor trunk posture, pelvic drop, and hip abduction weakness [15]. Most notably, in the orthopaedic trauma literature, the Squat and Smile test has been found to be a reliable surrogate for functional assessment of long bone fracture healing [16,17].

In the context of orthopaedic trauma, the squat offers a dynamic and sensitive measure of postinjury functional recovery. Patients recovering from tibial plateau fractures, distal femur fractures, or ankle fractures often experience persistent impairments in squat depth, control, and symmetry, even months after achieving full weight-bearing status. Biomechanical analysis has demonstrated that squat movement patterns in trauma patients differ significantly from those of uninjured controls, with greater joint-loading asymmetries and compensatory movement strategies [18]. Functional deficits in these patients can persist despite normal radiographic findings and pain resolution, suggesting that traditional markers of healing may not fully capture comprehensive functional recovery. Incorporating the squat into post-trauma rehabilitation protocols may help identify patients who require additional neuromuscular retraining, thereby improving long-term outcomes and reducing the risk of reinjury.

Collectively, this evidence positions the squat as a powerful functional benchmark in clinical orthopaedic evaluation. The present study therefore describes the Six Steps to Squats protocol, a structured rehabilitation framework for patients recovering from isolated lower-extremity trauma.

Objectives

The objectives of this article are (1) to provide a detailed, reproducible description of the Six Steps to Squats rehabilitation and assessment protocol; (2) to evaluate the feasibility of implementing the protocol and its outcome assessments in a Level 1 trauma clinic; and (3) to report baseline squat mechanics and functional status in the first 12 enrolled patients. The primary research question of the overall pilot study, which is ongoing, is whether a structured, progressive squat-based rehabilitation program can improve functional outcomes in adult patients newly cleared for weight-bearing after isolated lower extremity trauma. A secondary research question is whether specific fracture patterns or surgical approaches influence early squat mechanics and rehabilitation response. We hypothesize that patients participating in the Six Steps to Squats protocol will demonstrate greater improvements in squat depth, joint flexion angles, and functional outcome scores than control patients at similar postoperative timelines. As enrollment and follow-up are ongoing, the present article is limited to the protocol description, feasibility evaluation, and baseline data; comparative effectiveness outcomes will be reported in a subsequent analysis once the target sample size has been reached.

## Materials and methods

This prospective cohort study was conducted at Detroit Medical Center, Detroit, Michigan, a Level 1 academic trauma center affiliated with Wayne State University. The study was approved by the Wayne State University Institutional Review Board (IRB-24-04-6736; approved November 11, 2025), and all participants provided written informed consent prior to enrollment. Patient enrollment began on November 11, 2025, and is ongoing.

Study design

This prospective, comparative pilot study investigates a structured rehabilitation program entitled the "Six Steps to Squats" protocol, which aims to improve functional outcomes in adult patients recovering from isolated lower extremity orthopaedic trauma. The methodology outlined here is designed to allow reproducibility across clinical and research settings. The study is being conducted at an academic orthopaedic Level 1 trauma center, with approval from the institutional review board, and involves both a control and an experimental cohort. The present article reports the study protocol, feasibility of its administration, and baseline assessment data; longitudinal and comparative outcomes will be reported at study completion.

Patient selection and eligibility criteria

Eligible patients are adults aged 18 years or older who have undergone surgery for a single lower extremity fracture involving the femur, tibia, ankle, or foot. To be included in the experimental group, patients must have been designated weight-bearing as tolerated (WBAT) within the last 1 to 2 months. Control group patients are those who have been WBAT for a period ranging from 4 to 12 months. By design, no patients are enrolled between 2 and 4 months after WBAT designation; this interval separates the newly cleared experimental cohort from the established recovery control cohort. Exclusion criteria include a history of any prior orthopaedic surgery involving either lower extremity, multiple simultaneous lower extremity injuries, inflammatory arthritis, paraplegia, prior arthroplasty, a BMI greater than 45, or more than three active medical comorbidities. These inclusion and exclusion criteria are designed to reduce confounding variables related to comorbidity burden, chronic joint disease, or recovery potential.

Intervention

The intervention group receives the Six Steps to Squats rehabilitation program. Patients are instructed in the rehabilitation program and followed thereafter during their postoperative course to evaluate their progression. The control group undergoes postoperative rehabilitation without additional squat-based rehabilitation. Both groups undergo continued routine visits with their orthopaedic traumatologist and receive identical management in terms of physical therapy prescription, restrictions, and any other treatment.

Rehabilitation protocol

The Six Steps to Squats protocol, developed by the authors, is a progressive rehabilitation framework comprising six sequential phases, each designed to target key components of mobility, strength, and proprioception (Table 1). The protocol was designed in conjunction with physical therapists and orthopaedists, with the ultimate goal of achieving a full-flexion, unassisted squat. The individual exercises and progression criteria were adapted from established squat biomechanics and range-of-motion literature [2,3,6,19,20]. The progressive stages are intended to be self-paced by the patient and to allow self-assessment before advancement to the next step. The protocol does not utilize any specialized equipment, thereby improving accessibility. A patient guide with QR-coded links to instructional videos for every exercise supports correct, home-based performance.

**Table 1:** Six Steps to Squats Protocol Dynamic exercises are performed for two to three sets of 10 repetitions and static stretches for three 30-second holds per side or as comfortable, once daily. ROM, range of motion.

Step	Focus	Exercises	Graduation
1. Ankle mobility	Ankle range of motion and dorsiflexion	Passive wall calf stretches, calf raises, tibialis anterior raises	Calf raise with descent below the forefoot and full ascent
2. Hamstring mobility	Hip and knee mobility	Elephant walks, standing hamstring stretches	Ability to touch toes with knees straight
3. Passive deep squat	Passive hip, knee, ankle ROM	Rag pulls, rock into squats	Maximal flexion of the hip, knee, and ankle with a rag pull
4. Elevated split squat	Single leg hip, knee, and ankle active and passive ROM	Descend into deep flexion, ascend to standing	Maximal flexion with thigh resting on calf, unassisted descent and ascent
5. Squat descent	Both legs: hip, knee, ankle, maximal eccentric active ROM	Assisted squat descent, unassisted squat descent	Maximal flexion with thigh resting on calf and heel planted, unassisted descent
6. Squat descent + ascent	Both legs: hip, knee, ankle, maximal eccentric and concentric active ROM	Assisted squat descent and ascent, unassisted squat descent and ascent	Maximal flexion with thigh resting on calf and heel planted, unassisted ascent

Exercise prescription

To support replication, dosing was standardized across patients. Unless otherwise noted, dynamic mobility and strengthening movements (calf raises, tibialis anterior raises, elephant walks, rag pulls, rock-into-squat drills, elevated split squats, and squat descent and ascent repetitions) are performed for two to three sets of 10 repetitions per session, and static stretches (passive wall calf stretch and standing hamstring stretch) are held for 30 seconds and repeated three times per side. Patients complete one session daily, lasting approximately 10 to 30 minutes depending on the step reached. Repetitions are performed slowly and with control through the maximum comfortable ROM; achieving range with proper form is prioritized over repetition count, and patients are instructed to stop short of sharp pain.

Step 1 focuses on ankle ROM, particularly hyperdorsiflexion, as limited dorsiflexion is the most common cause of limited squat descent, and ankle stiffness is a common complication after lower extremity trauma [19,20]. This phase includes passive wall calf stretches and active calf raises to address a tight heel cord. Tibialis anterior raises are also included to develop dynamic dorsiflexion strength in the ankle, which will be necessary for eccentric squat descent. The ankle mobility exercises also provide a degree of benefit to the knee joint by improving ROM and strength of the gastrocsoleus complex. Competency in this phase, assessed either by research staff or self-reported by the patient, is achieved by complete descent and ascent during an active calf raise. Patients then advance to Step 2, which addresses knee and hip mobility through hamstring flexibility, as tight hamstrings limit hip flexion during the squat movement, especially in males [19,20]. This stage uses dynamic exercises such as elephant walks and static standing hamstring stretches to lengthen the hamstring tendon. Step 3 attempts to achieve full passive ROM of the hips, knees, and ankles through passive deep-squat range-of-motion drills, including rag pulls and rock-into-squat movements. Successful completion of this step entails the ability to achieve full passive ROM.

In Step 4, patients progress to single-leg full active ROM at the hip, knee, and ankle. This step consists solely of the single-leg elevated split squat exercise. Patients first focus on achieving maximal unilateral flexion with descent only, using assistance if necessary, and eventually progress to completing the single-leg split squat unassisted with maximal ROM. Next, Step 5 progresses to assisted and unassisted deep squat descent only, emphasizing both depth and form before progression to the final phase. Finally, Step 6 incorporates ascent, both assisted and unassisted, completing the full-flexion squat with maximal ROM of the hips, knees, and ankles. Maximal ROM is indicated by the thigh resting on the calf and the heel planted on the ground.

Patients are instructed to perform the exercises daily, progressing from one step to the next based on comfort, form, and ROM rather than following fixed timelines. The exercise program is additive; patients continue to perform exercises from earlier steps while advancing to subsequent steps. Emphasis is placed on mastering each level of mobility and control before introducing strength elements or advancing to the next phase. This approach allows patients with varying levels of recovery to progress at a self-paced, safe rate while ensuring that movements are performed with proper form to minimize compensation or reinjury. 

Supervision, safety, and adherence monitoring

All baseline and follow-up squat assessments are performed in the clinic under the direct supervision of trained research staff, with a stable support surface within reach of the patient and a practice attempt provided before formal measurement. The home exercise program itself is performed without direct supervision; however, several provisions were made to monitor and correct exercise performance. At enrollment, research staff demonstrate each exercise and observe the patient performing the exercises of their current step, providing corrective feedback on form. Patients receive an illustrated guide with QR-coded links to instructional videos for every exercise. At each monthly clinic visit, staff observe the patient performing the exercises of their current step and review technique before advancement is approved. Biweekly telephone calls are used to reinforce adherence, answer technique questions, and document pain, adverse events, and barriers to exercise.

Patients are given explicit safety instructions: exercises should be performed near a wall, countertop, or other stable support that can be grasped at any time; supportive non-slip footwear, or bare feet on a non-slip surface, should be used; movements should remain within a pain-free or minimally uncomfortable range; and any sharp pain, new swelling, sensation of instability, or fall should prompt cessation of the program and contact with the study team. Progression to the next step requires meeting the graduation criterion for the current step (Table 1), confirmed either by research staff at a clinic visit or by patient self-assessment against the same criterion.

Data collection

At the initial clinic visit, data collection began with informed consent. Patients then completed the validated general health and functional status questionnaire (SF-36) [21] and the 10-item squat questionnaire using a clinic iPad or their mobile phone. Both questionnaires were administered before the physical squat assessment so that responses reflected each patient's perception of their abilities prior to formal testing. A standardized baseline squat assessment followed: patients were given a practice attempt, followed by a formal evaluation of their maximum assisted and unassisted squat depth. Photographs were taken at the point of maximal flexion using a lateral view parallel to the ground for subsequent angle measurement.

Hip, knee, and ankle flexion angles were measured digitally with DetroitBoneSetter.com. Assisted squats involved the use of upper extremity support (e.g., a table or wall), with heel lifts added as needed to facilitate optimal depth. Squat angle data were recorded in a secure research database.

Demographic data, including age, BMI, comorbidities, injury type, and time since WBAT designation, were also recorded at enrollment. The control group followed the same initial visit procedure, except that control patients did not receive the rehabilitation protocol and were not scheduled for protocol follow-up. For experimental patients, follow-up visits occur monthly in the clinic. During each follow-up visit, squat assessments are repeated using the same imaging and measurement methods, and the SF-36 is re-administered at three-month intervals. Research staff also conduct biweekly phone calls to monitor progress, reinforce adherence, and document any adverse events, pain, or barriers to exercise. These qualitative notes are stored in patient logs. A SPIRIT (Standard Protocol Items: Recommendations for Interventional Trials) timetable adapted from the SPIRIT 2013 Statement illustrates the study design, enrollment, interventions, and assessment time points (Table 2) [22]. 

**Table 2:** SPIRIT Timetable SPIRIT timetable adapted from the SPIRIT 2013 statement illustrating the study design, enrollment, interventions, and assessment time points [22]. An "X" indicates the time point at which the listed activity is performed once. Arrows (→) indicate that the Six Steps to Squats intervention is delivered continuously from the baseline assessment through study close-out, rather than at a single time point. SF-36 PCS: 36-Item Short Form Health Survey Physical Component Summary, SPIRIT: Standard Protocol Items: Recommendations for Interventional Trials.

	Study period				
Timepoint	Enrollment (−2 to −1 days)	Allocation (T0)	Baseline assessment (T0)	Follow-ups (intervention only, monthly)	Close-out (final analysis)
Enrollment					
Eligibility screen	X				
Study information	X				
Informed consent	X				
Allocation		X			
Interventions					
Six Steps to Squats protocol (intervention)			⟶ ⟶ ⟶ ⟶	⟶ ⟶ ⟶ ⟶	⟶
Control group (usual care)			X		
Assessments					
Demographics		X			
Squat angles (hip, knee, ankle)			X	X	
Ability to rise from a squat			X	X	
SF-36 PCS			X	X	X
Squat questionnaire (10 items)			X	X	X
Adverse events, pain, barriers				X	X
Subgroup analysis					X

The primary outcomes of this study are squat performance, assessed through maximal hip, knee, and ankle flexion angles during both assisted and unassisted squats, and functional status, measured using the SF-36 Physical Component Summary score. Secondary outcomes include the ability to rise from an unassisted squat, subgroup differences based on fracture type and surgical technique, responses to a 10-item patient-reported squat questionnaire developed by the authors (Table 3), and documentation of adverse events, pain levels, and barriers to exercise encountered during rehabilitation. 

**Table 3:** Author-Developed 10-Item Patient-Reported Squat Questionnaire

Questions
Are you able to DESCEND to a deep squat (otherwise known as Indian squat, Slav squat, or full bend squat)? (Yes Fully; Yes Partially; No)
Are you able to RISE from a deep squat? (Yes Fully; Yes Partially; No)
Do you have PAIN in your HIPS when completing a squat? (No; Yes, One Hip; Yes, Both Hips)
Do you have PAIN in your KNEES when completing a squat? (No; Yes, One Hip; Yes, Both Hips)
Do you have PAIN in your ANKLES when completing a squat? (No; Yes, One Hip; Yes, Both Hips)
Do you have STIFFNESS in your HIPS when completing a squat? (No; Yes, One Hip; Yes, Both Hips)
Do you have STIFFNESS in your KNEES when completing a squat? (No; Yes, One Hip; Yes, Both Hips)
Do you have STIFFNESS in your ANKLES when completing a squat? (No; Yes, One Hip; Yes, Both Hips)
Are you currently participating in any sport, athletic training, or any other regular physical activity involving squats? (No; Yes)
Are you currently doing formal physical therapy? (No, 1-2 sessions a week, 3-4 sessions per week)

Demographic and injury-related variables (age, sex, BMI, comorbidities, fracture type, surgical treatment, and time since WBAT) were recorded at enrollment. Adverse events, pain levels, and barriers to exercise are documented during follow-up. Qualitative data are gathered through self-reported patient questionnaires. Initial questionnaires included the validated SF-36 Physical Component Summary, as well as an additional 10-item questionnaire developed to assess subjective perceptions of squat ability (Table 3); as noted above, both were completed before the physical squat assessment.

Statistical analysis

Given the absence of prior data on squat-based rehabilitation in trauma populations, the sample size calculation is based on an expected clinically meaningful difference in knee flexion angle of 10° (SD 12°) between groups at three months. To achieve 80% power with an alpha of 0.05, 25 participants per group are required. Accounting for an anticipated 20% to 30% attrition rate, a total sample size of 70 patients (35 per group) is targeted. For primary outcomes, changes in joint flexion angles and functional outcomes will be analyzed using repeated-measures ANOVA or mixed-effects regression models, adjusting for age, sex, BMI, fracture type, and time since WBAT. SF-36 outcomes will be analyzed using linear regression, with significance thresholds adjusted for multiple comparisons. Secondary analyses will compare subgroups by fracture type (e.g., ankle fractures vs. femur fractures). Intention-to-treat analysis will be performed alongside per-protocol analysis to account for adherence. Missing data will be addressed using multiple imputation methods. Qualitative findings will be integrated with quantitative outcomes to provide a comprehensive assessment of feasibility and effectiveness. These comparative analyses pertain to the completed trial; the present article reports descriptive baseline statistics only, and no between-group hypothesis testing was performed on the data presented here.

## Results

Enrollment began on November 11, 2025, and is ongoing. All measurements reported in this article were collected at each patient's single baseline study visit, before any patient had begun the Six Steps to Squats protocol. For experimental patients, the baseline visit occurred within 2 months of WBAT designation; for control patients, it occurred 4 to 12 months after WBAT designation. At the time of this analysis, a total of 12 patients were enrolled: seven in the control group and five in the experimental group.

**Figure 1:**
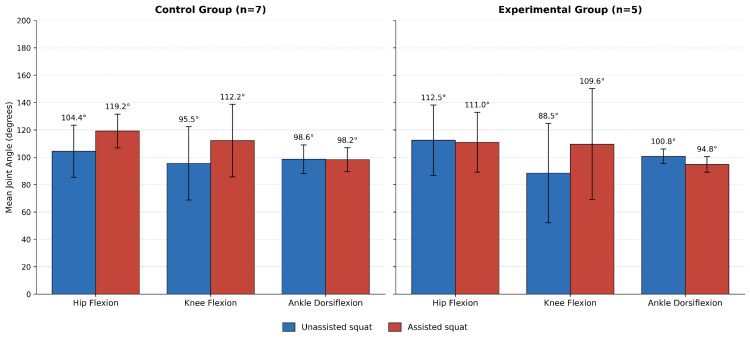
Baseline Joint Flexion Angles: Control vs Experimental Baseline joint flexion angles during unassisted and assisted squat assessments in control (n = 7) and experimental (n = 5) groups. Mean maximum hip flexion, knee flexion, and ankle dorsiflexion angles are shown for each group, with blue bars representing unassisted squats and red bars representing assisted squats. Error bars indicate ± 1 standard deviation. Values above each bar denote the group means in degrees.

Control group patients demonstrated a mean unassisted hip flexion angle of 104.4 ± 19.1 degrees, unassisted knee flexion of 95.5 ± 26.8 degrees, and unassisted ankle dorsiflexion of 98.6 ± 10.5 degrees. In comparison, the experimental group demonstrated slightly higher mean unassisted hip flexion at 112.5 ± 25.7 degrees and ankle dorsiflexion at 100.8 ± 5.3 degrees, but lower mean knee flexion at 88.5 ± 36.3 degrees. During assisted squats, control group participants averaged 119.2 ± 12.4 degrees for hip flexion, 112.2 ± 26.5 degrees for knee flexion, and 98.2 ± 8.8 degrees for ankle dorsiflexion. The experimental group averaged 111.0 ± 21.9 degrees, 109.6 ± 40.6 degrees, and 94.8 ± 5.7 degrees for assisted hip, knee, and ankle angles, respectively (Figure 1). Demographic information is displayed in Table 4. 

**Table 4:** Baseline Demographic and Clinical Characteristics of Enrolled Patients (N = 12) *BMI was not recorded for one control patient; the mean reflects the six patients with available data. †By design, the experimental group is enrolled within 2 months of WBAT designation, and the control group between 4 and 12 months after WBAT designation; therefore, no enrolled patients fall within the interval of greater than 2 and less than 4 months. SD, standard deviation; WBAT, weight-bearing as tolerated.

Characteristics	Control (n = 7)	Experimental (n = 5)
Age, years		
Mean (SD)	50.0 (16.2)	44.4 (13.4)
Range	28-74	30-58
Sex, n (%)		
Male	4 (57.1)	2 (40.0)
Female	3 (42.9)	3 (60.0)
Race, n (%)		
Black/African American	4 (57.1)	5 (100.0)
White/Caucasian	3 (42.9)	0 (0.0)
Body mass index, kg/m²		
Mean (SD)	29.1 (3.3)*	28.0 (7.6)
Range	26.5-35.1	19.3-39.3
Smoking status, n (%)		
Never smoker	5 (71.4)	2 (40.0)
Current smoker	1 (14.3)	1 (20.0)
Former smoker	0 (0.0)	2 (40.0)
Not recorded	1 (14.3)	0 (0.0)
Comorbidities, n (%)		
Asthma	1 (14.3)	1 (20.0)
None reported	6 (85.7)	4 (80.0)
Fracture location, n (%)		
Femur	1 (14.3)	1 (20.0)
Tibia (including plateau)	2 (28.6)	2 (40.0)
Ankle/foot	3 (42.9)	2 (40.0)
Acetabulum/hip	1 (14.3)	0 (0.0)
Surgical procedure, n (%)		
Intramedullary nail (IMN)	1 (14.3)	1 (20.0)
Open reduction internal fixation (ORIF)	6 (85.7)	4 (80.0)
Time since WBAT designation, n (%)		
≤2 months	0 (0.0)	5 (100.0)
>2 to <4 months†	0 (0.0)	0 (0.0)
4 to 6 months	3 (42.9)	0 (0.0)
>6 months	4 (57.1)	0 (0.0)

All 12 patients completed the full baseline assessment, including both questionnaires, the supervised squat evaluation, and photographic documentation, within a single routine clinic visit. Digital angle measurement was obtainable at all three joints in every patient, and no adverse events, falls, or episodes of significant pain occurred during testing.

All patients were able to rise from their deepest unassisted squat descent at the initial visit. Notably, the experimental group patients had not yet begun the Six Steps to Squats protocol at the time of data collection; they had only recently been designated WBAT and qualified for the intervention based on the inclusion and exclusion criteria. As such, the observed measurements reflect their baseline status prior to any intervention.

Preliminary subgroup analysis suggested that patients with bimalleolar ankle fractures repaired with open reduction and internal fixation may have lower joint flexion angles across multiple planes. This observation, while not yet statistically evaluated, may point to greater mechanical limitations in this subgroup.

These early results provide a baseline snapshot of squat mechanics in patients recently cleared for WBAT following lower extremity trauma. Future follow-up visits will assess progression in ROM and functional outcomes following engagement with the rehabilitation protocol.

Data management and reproducibility

All patient data are collected in compliance with HIPAA regulations. Protocol fidelity is maintained through standardized training of research personnel and access to digital instructional resources for patients. The methodology is designed to function effectively within busy clinical settings, supporting its potential scalability for broader use in orthopaedic rehabilitation protocols. By providing a structured, evidence-informed framework, this study establishes a foundation for validating the squat as a functional benchmark across diverse orthopaedic populations.

## Discussion

This study introduces a structured, reproducible squat-based rehabilitation protocol designed for orthopaedic trauma patients newly cleared for WBAT and reports the feasibility and baseline data of its ongoing pilot evaluation. While traditional metrics in orthopaedic recovery often emphasize static measurements such as ROM or time to ambulation, these do not always capture the integrated neuromuscular control, coordination, and strength required for real-world function [1,7]. The squat, in contrast, is a simple but dynamic, full-body movement that reflects a patient's ability to engage their lower extremities in a weight-bearing, functional manner [2,3]. Our preliminary results demonstrate wide variability in joint flexion angles and functional capacity across patients early in the recovery timeline, which reinforces the need for a standard functional assessment tool in this population.

The inclusion of patients at their initial post-WBAT visit allowed for a clean baseline assessment, free from the confounding effects of prior structured training. Interestingly, unassisted squat performance in some experimental group patients exceeded that of the control group; however, these differences likely reflect injury type and individual variability [19]. Notably, patients with bimalleolar open reduction and internal fixation (ORIF) demonstrated consistently lower hip, knee, and ankle flexion angles, suggesting that fracture pattern and surgical approach may significantly affect early squat mechanics [18,20]. This trend, although preliminary, supports the notion that squat-based evaluation may help stratify functional risk early in recovery.

Our baseline observations are consistent with prior work on squat-based assessment in related populations. Kim et al. demonstrated that ankle dorsiflexion and hip ROM are key determinants of squat depth in healthy adults [19], which aligns with the reduced multi-planar flexion we observed in patients with bimalleolar ankle fractures treated with ORIF, a group in which postoperative ankle stiffness and arthrofibrosis are well described [20]. The persistence of functional limitation in our control group, assessed 4 to 12 months after WBAT designation, parallels the findings of Kröger et al., who reported that gait and functional deficits remain measurable six months after tibial shaft fracture despite progression of healing [18]. In ACL reconstruction populations, Hall et al. found that approximately 45% of patients failed a single-leg squat assessment six months postoperatively despite completing standard rehabilitation [9], reinforcing the value of squat-based testing for detecting residual deficits that time-based criteria miss. Structured squat-focused training has improved functional outcomes in knee osteoarthritis [13], and sit-to-stand testing correlates with independence and real-world mobility in older adults [14]. Finally, the Squat and Smile test has been validated as a pragmatic surrogate for long bone fracture healing in resource-limited settings [16,17]. The Six Steps to Squats protocol builds on this body of work by combining a progressive, equipment-free training program with quantitative, multi-joint angle measurement specifically for the orthopaedic trauma population.

Preliminary feasibility of this protocol in a clinical setting is supported by several concrete observations from the baseline cohort: all 12 enrolled patients completed the full assessment battery, including questionnaires, supervised squat testing, and photographic angle measurement, within a single routine clinic visit; digital flexion angles were obtainable at all three joints in every patient; and no adverse events occurred during testing. The simplicity of the movement and the training protocol allows patients to self-evaluate their progress as they improve their ROM. The photographic documentation of squat performance also offers potential for visual feedback to patients and providers alike, reinforcing adherence and guiding progression. Additionally, the multi-joint nature of the squat enables a holistic perspective on patient recovery, which is often not captured by single-plane assessments or general activity reports. Formal feasibility benchmarks for the complete trial, including recruitment rate, protocol adherence, and retention, will be reported at study completion.

Strengths

This study has several strengths. To our knowledge, the Six Steps to Squats protocol is the first standardized, progressive squat-based rehabilitation and assessment framework designed specifically for orthopaedic trauma patients, addressing a gap in the literature in which squat assessment has been validated in ACL reconstruction, osteoarthritis, and global health settings but not systematically in trauma [9,13,16,17]. The protocol requires no specialized equipment, supporting accessibility and home-based use across diverse patient populations. Outcome assessment combines objective digital measurement of flexion angles at three joints with a validated general health instrument (SF-36) and a condition-specific patient-reported questionnaire, providing a multidimensional picture of recovery. The study design follows SPIRIT guidance for protocol reporting [22]. Enrollment of experimental patients at the initial post-WBAT visit provides a clean baseline free from confounding by prior structured training, and the prespecified sample size calculation and analysis plan position the ongoing trial to evaluate effectiveness rigorously.

Limitations

Several limitations of this study warrant consideration. First, the small sample size (N = 12) reflects the preliminary nature of this cohort and represents only a fraction of the planned 70-patient sample; it does not permit formal between-group comparisons, and results should therefore be interpreted as descriptive and hypothesis-generating rather than confirmatory. Second, group sizes are unequal at this stage (seven control and five experimental patients), and allocation is based on time since WBAT rather than randomization, which limits comparability between groups. Third, this is a single-center study conducted at a Level 1 academic trauma center, which may limit generalizability to community or non-trauma orthopaedic settings. Fourth, the heterogeneity of fracture patterns, surgical approaches, and time since WBAT designation across enrolled patients introduces clinical variability that a larger cohort will be better positioned to address through stratified analysis. Fifth, the home exercise program is performed without direct supervision between clinic visits; although observed performance at monthly visits, biweekly telephone calls, and instructional video resources mitigate this limitation, exercise fidelity at home cannot be fully verified. Finally, as enrollment is ongoing, longitudinal outcomes following implementation of the Six Steps to Squats protocol are not yet available; future analyses will report functional trajectories and patient-reported outcomes as the cohort matures.

As the study progresses, further data collection will allow for longitudinal tracking of improvements within the experimental group following implementation of the Six Steps to Squats protocol. Comparisons with the control group will provide insights into whether a structured, squat-focused rehabilitation regimen yields superior outcomes in functional recovery, squat biomechanics, and potentially patient-reported metrics. These outcomes may inform future protocols for orthopaedic trauma patients, helping to define a new standard for functional rehabilitation benchmarks.

## Conclusions

This article describes the design, rationale, and baseline implementation of the Six Steps to Squats protocol, a structured, progressive squat-based rehabilitation and assessment framework for patients recovering from isolated lower extremity trauma. The squat is a validated, functionally relevant movement pattern that reflects integrated joint mobility, neuromuscular control, and real-world physical capacity. In the first 12 enrolled patients, the protocol's assessments proved feasible to administer within routine trauma clinic visits, and baseline data revealed wide variability in squat mechanics early in recovery, with potential subgroup differences such as reduced multi-planar flexion after bimalleolar ORIF. As these data are baseline measurements from an ongoing pilot study with a planned sample of 70 patients, no conclusions regarding the effectiveness of the protocol can be drawn at this time. Continued enrollment and follow-up will determine the protocol's impact on joint flexion, patient-reported outcomes, and functional recovery and will clarify its role in post-trauma rehabilitation. If the ongoing trial supports its efficacy, the Six Steps to Squats protocol may serve as a reproducible model for clinical application and research in orthopaedic functional recovery.
